# Immunopathology of Antineutrophil Cytoplasmic Antibody (ANCA)-Associated Vasculitis

**DOI:** 10.3390/ijms26136065

**Published:** 2025-06-24

**Authors:** Katarzyna Aleksandra Lisowska, Anna Wardowska

**Affiliations:** Department of Pathophysiology, Medical University of Gdańsk, 80-210 Gdańsk, Poland

**Keywords:** ANCA-associated vasculitis, AAV, GPA, MPA, EGPA, classification, immunopathology, immune cells, genetic, epigenetic

## Abstract

Antineutrophil cytoplasmic antibody (ANCA)-associated vasculitis (AAV) constitutes a group of rare diseases characterized by autoimmune-associated inflammation and vessel damage. Based on the clinical manifestations and involvement of immune components, three disease syndromes are distinguished: granulomatosis with polyangiitis (GPA), microscopic polyangiitis (MPA), and eosinophilic granulomatosis with polyangiitis (EGPA). In this review, we present the current data on the epidemiology, the clinical manifestations of each syndrome, and the most up-to-date classification criteria. The role of the underlying genetic and epigenetic abnormalities, as well as their interplay, is described. The immunological diversification of AAV is also described, with a focus on the immune cell dysfunctions detected in patients. In conclusion, we emphasize the urgent need to unravel the sophisticated mechanisms of this disease, which would enable the development of new, effective therapeutic strategies.

## 1. Introduction

Antineutrophil cytoplasmic antibody (ANCA)-associated vasculitis (AAV) is a group of rare disorders [1,2] associated with the inflammation and damage of predominantly small- and medium-sized vessels [3,4]. Based on the clinical picture, AAV is divided into three disease syndromes: granulomatosis with polyangiitis (GPA), formerly known as Wegener’s granulomatosis; microscopic polyangiitis (MPA); and eosinophilic GPA (EGPA), formerly known as Churg–Strauss syndrome [3]. Each subtype has unique clinical and pathological features. MPA typically presents with glomerulonephritis and pulmonary capillaritis [4], while GPA and EGPA are predominantly characterized by respiratory tract involvement. Notably, GPA and EGPA are further distinguished by the presence of granulomatous inflammation [5]. The AAV pathogenesis is highly complex and results from a combination of overlapping factors that lead to immune tolerance breakdown. The multifactorial background of vasculitis includes genetic predisposition, environmental factors, sex hormones, and immunological mechanisms.

Genetic factors, such as variations in the HLA (human leukocyte antigen) or PRTN3 (proteinase 3) genes, play a crucial role [6]. However, epigenetic factors also contribute significantly to disease susceptibility. Among environmental factors, ultraviolet (UV) radiation [7], smoking [8], pesticides, fertilizers [9], and some other occupational exposures have been implicated as potential triggers.

This review aims to explore the mechanisms underlying the clinical manifestation of AAV, with a particular focus on genetic and cellular processes. In recent years, epigenetic mechanisms have been studied extensively, especially regarding their role in linking altered epigenetic patterns to pathological processes in the human body [10]. The dysregulation of these mechanisms can lead to changes in gene expression, disrupting immune homeostasis and promoting pathological autoimmune processes. Finally, we discuss the mechanisms contributing to the development of vasculitis cases without ANCA, highlighting the heterogeneity of these conditions and the need for further research.

## 2. Epidemiology

Antineutrophil cytoplasmic antibody (ANCA)-associated vasculitis (AAV) is a relatively rare disease [1,2]. It is classified as an orphan disease under the number ORPHA:156152 on Orphanet [11]. However, the incidence of AAV has increased over time, plausibly due to the improvements in ANCA testing, disease classification, and clinical recognition [12,13]. Presently, AAV has an estimated prevalence of 200 cases per million people [1,2,14,15] and an incidence of about 20 per million person-years in Europe and North America [14,15].

According to the latest meta-analysis by Redondo-Rodriguez et al. [14], the highest incidence of AAV is observed in Minnesota and the lowest in Turkey. The AAV prevalence is also the highest in Minnesota. The pooled incidence and prevalence are greater for GPA than for MPA and EGPA. According to Watts et al. [15], MPA is the most common in Southern Europe and Japan, while GPA is more common in other populations. These studies show that there are differences regarding the variability between the northern and southern hemispheres and between continents, thus highlighting the ethnic and genetic differences. Some studies suggest that the incidence and prevalence may depend on the latitude. In 2009, Gatenby et al. [7] demonstrated that the incidence of AAV, especially GPA and EGPA, increases with increasing latitude and decreasing ambient UV radiation. A similar observation was made by Li et al. [1], which would suggest that low UV radiation exposure, possibly by influencing vitamin D levels, could, combined with other factors, stimulate AAV development [16].

In Poland, in 2014, a register of primary vasculitis was established as part of the Scientific Consortium of the Polish Vasculitis Registry (POLVAS). It is one of several such initiatives in Europe. The Consortium aims to collect epidemiological and clinical data on all forms of primary and secondary vasculitis, both retrospectively and prospectively. It conducted a multicenter retrospective study of all adult patients diagnosed with AAV between 1990 and 2016, identifying 625 AAV adults. It was found that 66.7% of patients from the POLVAS registry were diagnosed with GPA, 17.0% with MPA, and 16.3% with EGPA [17].

## 3. Clinical Manifestation

The clinical manifestations of AAV largely depend on the vascular bed affected, with the lungs and kidneys being the most common sites of involvement [18]. The disease can be limited to one organ at the time of presentation or involve multiple organs. Kidney involvement in AAV is widespread and can follow a slow or rapid progressive disease course, in time leading to dialysis.

All three disorders share symptoms of systemic inflammation, including weight loss, malaise, and fatigue. Approximately 50% of AAV patients also present arthralgia and myalgia [19]. Although various organs may be affected by AAV, and there is a significant clinical overlap between these diseases, each AAV demonstrates distinct pathological profiles. MPA typically presents with glomerulonephritis and pulmonary capillaritis, leading to interstitial pneumonitis and lung hemorrhage, which often worsens rapidly. Skin, nerves, and gastrointestinal tract involvement is common [19].

GPA and EGPA are characterized by a predominant respiratory tract involvement and can be further distinguished by granulomatous inflammation, primarily neutrophilic in GPA and eosinophilic in EGPA [5]. Patients frequently report symptoms of chronic rhinitis, sinusitis, or laryngitis [20]. Asthma is a typical early feature of EGPA [21]. Some studies have shown that heart and neurological involvement is highly prevalent in EGPA patients [17]. Meanwhile, 10–60% of GPA patients suffer from hyperplastic gingivitis and frequently experience ocular symptoms, including uveitis, ocular ulcers, episcleritis, and scleritis [19].

## 4. AAV Classification

The most widely used systemic vasculitis classification system was established at the 2012 International Chapel Hill Consensus Conference (CHCC). This system stratified vasculitis according to vessel size [3,22]. According to this system, AAV was then divided into three clinical diseases: granulomatosis with polyangiitis (GPA), microscopic polyangiitis (MPA), and eosinophilic GPA (EGPA). These clinical phenotypes are characterized by vasculitic necrotizing lesions that predominantly affect arterioles, small arteries, capillaries, and venules [23,24].

The current classification criteria for GPA, MPA, and EGPA, developed by the ACR/EULAR (American College of Rheumatology/European Alliance of Associations for Rheumatology), were published in 2022 [25,26,27]. These criteria were the result of many years of collaborative work with rheumatologists, nephrologists, and immunologists, leading to the creation of weighted criteria for MPA [26], GPA [25], and EGPA [27].

In addition to the clinical symptoms and histopathological findings, ANCA antibodies are a helpful tool in recognizing the specific type of AAV (Table 1). ANCAs primarily target myeloperoxidase (MPO-ANCA) or proteinase 3 (PR3-ANCA) [28,29]. MPO-ANCAs may also react with other enzymes such as cathepsin G, elastase, lactoferrin, and lysozyme [30]. In immunofluorescence staining, PR3-ANCA shows a cytoplasmic pattern, hence the term c-ANCAs, while MPO-ANCA displays a perinuclear pattern (p-ANCA) [31]. c-ANCAs are mainly associated with GPA (75%) or renal-limited vasculitis, while p-ANCAs are more frequently found in MPA (60%). Several studies have shown that ANCAs, especially MPO-ANCA, are also present in approximately 50% of EGPA patients [3,28,32]. Despite this, the authors of the ACR/EULAR criteria did not include MPO-ANCA or PR3-ANCA positivity in the final criteria of EGPA, because these antibodies are significantly more prevalent in MPA and GPA [27]. Recent studies suggest that patients can also be positive for both types of antibodies [33]. These patients exhibit a combination of clinical characteristics of patients with both antibody types but are generally more similar to patients with MPO-ANCA.

Though ANCAs are crucial for the diagnosis of AAV, there are cases where antibodies are not detected [34]. Approximately 10% of GPA patients are ANCA-negative, mainly those with the disease limited to the upper and lower respiratory tract [35]. A similar case concerns MPA; ANCA-negative MPA patients usually present only renal symptoms or a less severe systemic disease [36]. Moreover, both GPA and MPA may become ANCA-positive in the course of the disease, suggesting a dynamic relationship between the autoantibody presence and disease progression [37]. Only half of EGPA patients are ANCA-positive, with positivity often correlating with a more vasculitic phenotype (vasculitic EGPA), while ANCA-negative cases are more frequently associated with eosinophilic tissue infiltration (non-vasculitic EGPA) [38]. Therefore, vasculitic EGPA usually presents with kidney involvement that quickly progresses and an alveolar hemorrhage. Meanwhile, non-vasculitic EGPA is characterized by cardiac involvement, pleural infusion, and gastroenteritis.

These observations imply that ANCAs are not essential for the initiation or pathogenesis of AAV. Additionally, their absence does not exclude the diagnosis, underscoring the importance of clinical evaluation and complementary diagnostic tools such as histopathology and imaging.

## 5. Genetic Background of AAV

### 5.1. HLA Genes and Their Association with AAV

Familial AAV cases have been reported for GPA [39] and EGPA [40]. Additionally, overlapping syndromes, mainly between GPA and EGPA, have been described [41]. However, a putative hereditary factor has never been definitively demonstrated. Studies show that the relative familial risk (RR 1.56) for GPA [42,43] is similar to that for rheumatoid arthritis (RA) (RR 1.5–5.0) but lower than that for other immune-mediated diseases [44].

Several studies have shown associations between HLA (human leukocyte antigen) class II loci and AAV, with variations dependent on the ethnic group and geographic region. The association has been particularly noted with HLA-DPA1, HLA-DPB1, HLA-DQA1, HLA-DQB1, and HLA-DRB1 [45]. For example, HLA-DPB1*0401 alleles have been associated with GPA in German patients [46,47]. As for the Chinese population, HLA-DRB1*1202 has been associated with GPA, while HLA-DRB1*1101 has been associated with MPA [48]. In Japanese patients, MPA was associated with HLA-DRB1*0901 [49]. HLA-DRB1*07 was associated with EGPA in the Italian population [50] and the German population [51]. However, in German patients, a stronger correlation was found for HLA-DRB1*04 [51].

### 5.2. Role of Non-HLA Genes in AAV

Several non-HLA gene associations with AAV have been found, particularly genes involved in immune responses and ANCA generation. These include polymorphisms in CTLA4 (cytotoxic T lymphocyte-associated antigen-4), TLRs (Toll-like receptors), PTPN22 (protein tyrosine phosphatase, non-receptor type 22), SERPINA1 (serine protease inhibitor), and PRTN3 (proteinase 3) [6].

CTLA-4, a member of the CD28 receptor family, is a regulatory molecule expressed on the surface of T cells. It plays a significant role in maintaining peripheral tolerance by inhibiting autoreactive T cells, thus preventing autoimmune diseases [52]. Inhibiting T-cell activity is mediated through the binding with CD80 (B7-1) and CD86 (B7-2) present on antigen-presenting cells (APCs) [53]. Several studies have linked single-nucleotide polymorphisms (SNPs) in the CTLA4 gene to AAV, especially GPA. A British study found that the allele (A) of rs3087243 SNP has a protective effect against AAV [54]. Polymorphisms in exon 1 (+49) and 4 (CT60) were also associated with AAV in a separate British cohort [55].

TLRs, which are present on immune cells and are involved in pathogen recognition and antigen presentation, have been implicated to be associated with several autoimmune diseases, including AAV. Different TLRs recognize different pathogen-associated molecular patterns (PAMPs). Evidence suggests that TLR9, which recognizes unmethylated CpG motifs in bacterial nucleic acid, may be involved in AAV development. In a German study, four different SNPs in the TLR9 gene (rs352162, rs352140, rs352139, and rs5743836) were strongly associated with GPA [56]. While similar findings were observed for MPA, the associated SNPs had the contrary alleles. No association was found for EGPA. The authors could not confirm their results in Dutch and British AAV cohorts.

Polymorphisms in PTPN22, a gene encoding an intracellular tyrosine phosphatase that inhibits T-cell activation, are also linked with autoimmune diseases. The first study investigating the effect of SNPs in this gene revealed a strong association between rs2476601 and GPA, especially in ANCA-positive patients from the German population [57]. Similar results were later reported in British GPA and MPA patients [54]. In an Italian study, the 620 W SNP was found to be significantly associated with ANCA-positive GPA [58].

PRTN3, which encodes proteinase 3 (PR3), one of the main ANCA targets, is another gene linked to AAV. In Swedish patients, the A564G variation in the promoter region was found to be associated with GPA [59]. Additionally, in the Egyptian population, SNP rs62132295 and rs62132293 were significantly associated with AAV, especially PR3-AAV and GPA [60].

Finally, SERPINA1, which encodes α1-antitrypsin (α1-AT), the primary inhibitor of PR3, has been also implicated in AAV. Variants leading to reduced α1-AT function may result in persistent PR3 activity, promoting tissue damage and AAV development. Some studies showed that heterozygous patients for the Z variant (Glu342Lys) of SERPINA1 have an increased risk of developing GPA compared to the general population [61,62].

### 5.3. Insights from Genome-Wide Association Studies (GWASs)

Genome-wide association studies (GWASs) have provided insights into the genetic background of AAV, particularly GPA and MPA [63,64,65]. Several studies have confirmed associations between AAV and specific genetic loci, with a stronger correlation observed for antibody subtypes than for clinical manifestations. In one of the first GWAS studies, associations with GPA and MPA were identified at three loci: HLA-DPB1, SERPINA 1, and PRTN3 [64]. Among these, PR3-ANCA positivity emerged as the strongest factor linked to these SNPs. This finding highlighted the role of the immune system and genetic predisposition in shaping disease subtypes. The second study by Xie et al. [65] showed an association between the HLA-DPB1 and HLA-DPA1 loci and a novel locus, SEMA6A, particularly in GPA patients. As in the earliest studies, these associations were most potent in the PR3-ANCA-positive patients, emphasizing the distinct genetic signatures of the PR3-associated disease.

Interestingly, despite early hypotheses, GWAS failed to confirm the association between CTLA4 SNPs and AAV, suggesting a more limited role for this gene in disease susceptibility.

Further expanding this genetic framework, most recent GWAS studies identified a strong association between HLA-DQ and EGPA, particularly MPO-ANCA EGPA [63]. This highlights the heterogeneity within AAV subtypes and underscores the importance of integrating genetic data with clinical and serological profiles for improved disease classification and understanding.

In recent years, epigenetic mechanisms have been extensively studied, leading to a deeper understanding of the complex relationship between altered epigenetic patterns and pathological processes [10,66,67]. Epigenetics, broadly defined, refers to mechanisms that may alter the gene expression without changes in the DNA nucleotide sequence. Three essential modifications, collectively referred to as the epigenetic triad, form the foundation of epigenetics: DNA methylation, chromatin remodeling through histone modifications, and non-coding RNAs [68]. Dysregulated epigenetic mechanisms can disrupt gene expression and destabilize immune homeostasis, contributing to the development of autoimmune diseases [10,69].

The intricate interplay between these epigenetic mechanisms and their contributions to autoimmune pathogenesis is exemplified by studies on AAV [70,71]. Although the role of epigenetics in AAV remains relatively underexplored, initial findings highlight its significance in investigating epigenetic modifications in neutrophils from AAV patients and observed that the levels of the chromatin modification H3K27me3 were depleted at PRTN3 and MPO loci. This finding suggests that the epigenetic modifications of ANCA autoantigen-encoding genes may contribute to the inappropriate expression of PR3 and MPO in AAV. A complementary work by Yang et al. investigated the expression of proteins responsible for histone modification, euchromatic histone-lysine N-methyltransferase 1 and 2 (EHMT1, EHMT2), and male sex lethal 1 homolog and insulin growth factor (MSL1 and ING4) [72]. They found that EHMT1 and EHMT2 are associated with H3K9me2, responsible for gene silencing, and their expression in granulocytes from AAV patients is decreased compared with healthy people. Meanwhile, the expression of MSL1, associated with H4K16ac which activates genes, was overexpressed. Jones et al. [70] demonstrated that AAV patients with an active disease display the hypomethylation of MPO and PRTN3 genes, leading to the increased expression of these autoantigens, while, in remission, gene methylation increases.

In general, these works represent a first and significant step in linking the epigenetic modifications and disease phenotype in AAV. Unfortunately, there is too little research to precisely determine the importance of epigenetics in the development of AAV.

## 6. Immunopathomechanism in AAV

Immunologically, GPA and MPA are driven by aberrantly activated neutrophils, while EGPA is characterized by eosinophilic infiltration [5]. These cells are primed by specific autoantibodies targeted at neutrophil autoantigens—PR3 and MPO [28,29]. After reaching the vascular endothelium, activated cells release pro-inflammatory mediators. However, immunologically, AAVs are much more complex. Not only innate but also acquired response mechanisms are responsible for the development of the disease.

### 6.1. The Role of ANCA and Neutrophils

ANCA antigens, specifically PR3 and MPO, are primarily found in the granules of neutrophils. Eosinophils also express PR3 [73], together with eosinophil peroxidase (EPO), which shares a high structural similarity (69.8% amino acid) to MPO [74]. MPO and EPO are enzymes that catalyze the formation of hypochlorous acid, a potent chemical compound with strong antibacterial and antiviral properties [75]. PR3 is a serine protease enzyme responsible for degrading extracellular proteins, playing a role in immune defense and tissue remodeling [76].

Under healthy conditions, PR3 and MPO are stored in intracellular granules of neutrophils. However, in response to specific stimuli, these enzymes can be expressed on the surface of neutrophils [77] or monocytes [78]. The PR3 expression on neutrophils is bimodal with two distinct populations: one with a low and one with a high PR3 expression [78]. When neutrophils with a high PR3 expression are stimulated with PR3-ANCA, they generate higher amounts of superoxide, a reactive oxygen species (ROS), compared to cells with a lower PR3 expression. Neutrophils and monocytes can also express minimal surface MPO, with its expression increasing following cell mobilization [77,79].

**Figure 1:**
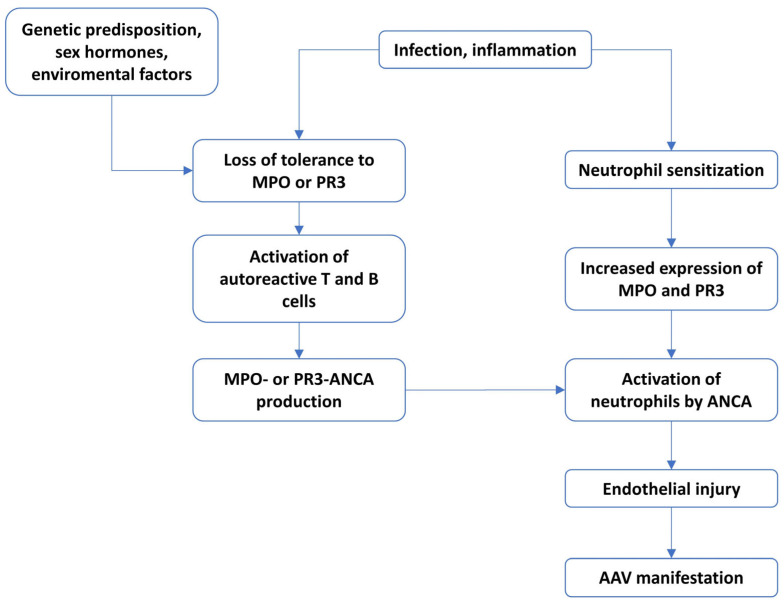
The course of AAV development.

For AAV to develop, there must first be the loss of the immunological tolerance of T and B cells towards MPO, which is necessary for the production of autoantibodies that play a role in vasculitis (Figure 1). Tan et al. showed that the deletion of autoreactive anti-MPO T cells in the thymus and the presence of peripheral regulatory T cells (Tregs) that suppress potentially autoreactive MPO-specific CD4+ T cells protect mice against glomerulonephritis (GN) [80]. MPO-ANCA-induced GN in mice is more aggressive in the presence of MPO-specific CD4+ T cells [81]. The role of PR3 is more complicated due to its presence on the surface of neutrophils. According to some studies, when non-obese diabetic (NOD) mice are injected with PR3, this results in the increased production of PR3-ANCA without inducing AAV manifestation, which suggests that the presence of antibodies alone is not sufficient to cause the disease [82]. Recently, Sharma et al. identified in the blood of AAV patients CD4+ T cells producing interferon-gamma (IFN-γ) in response to stimulation with PR3 [83].

ANCAs activate neutrophils and monocytes by engaging their Fc receptors after binding MPO or PR3 [84], or by Fab’2 binding MPO and PR3 expressed on their surface [85]. Additionally, the aberrant glycosylation of MPO- and PR3-specific ANCAs has been shown to enhance their ability to interact with activating Fc receptors on neutrophils, thereby increasing their immune-activating potential [86].

In vitro studies have demonstrated that ANCAs stimulate neutrophils to release ROS and undergo degranulation [87]. However, in response to ANCA binding, neutrophils can also release neutrophil extracellular traps (NETs), which are part of a defense mechanism against infections. In healthy conditions, NETs are released from activated neutrophils in response to pathogen-associated molecular patterns (PAMPs) or damage-associated molecular patterns (DAMPs) [88]. NETs are unique net-like structures composed of DNA strands, histones, and antimicrobial proteins such as MPO, cathepsin, and elastase [89], which are all autoantigens recognized by ANCA [30]. In vitro studies confirmed that ANCA-induced NETs are responsible for the damage of endothelial cells (ECs) [90,91] mediated through complement activation [91]. Moreover, in a mouse model, it was shown that PR3 and MPO from NETs can also be transferred to dendritic cells (DCs) [92]. The immunization of mice with these NET-loaded DCs led to ANCA production and subsequent kidney damage, suggesting a potential pathophysiological role for NETs in the development of AAV.

Kessenbrock et al. observed NET formation in the kidneys of patients with small-vessel vasculitis (SVV) and further demonstrated that ANCA-stimulated neutrophils release NETs containing PR3 and MPO [93]. Some authors demonstrated the presence of NETs in other organs affected by AAV, like the lungs [94], or the nervous system [95]. In healthy people, NET-DNA is processed by deoxyribonuclease (DNase) I [96]. NETs, mainly their remnants, are found in the blood of AAV patients [97]. At the same time, studies showed that the activity of DNase I is reduced in AAV patients which decreases NET degradation and makes PR3 and MPO available for ANCA [98].

At this point, it should be added that MPO-ANCA, which is so important for the pathogenesis of AAV, has also been found in other autoimmune diseases, including systemic lupus erythematosus (SLE) and Sjögren’s syndrome (SS) [99]. However, in the majority of those patients, ANCA did not recognize p-ANCA-related antigens, and their role in systemic autoimmune diseases is currently unknown [99]. Both types of ANCA were also shown in infective endocarditis, tuberculosis, primary sclerosing cholangitis, and interstitial lung diseases [100]. Moreover, studies showed that patients with idiopathic pulmonary fibrosis who were MPO-ANCA-positive can develop MPA [101]. Such observations suggest that the presence of ANCA in certain clinical situations may predict the development of AAV.

In vivo, AAV patients exhibit an increased percentage of polymorphonuclear leukocytes (PMNs) in their blood compared with healthy people [102]. Interestingly, there are no significant differences in the expression of certain surface antigens, such as CD88, CD11c, or CD62L, between patients and healthy controls. CD88 plays the role of a receptor for 5a, which is a protein fragment released from the cleavage of the complement component C5. C5a is an anaphylatoxin that causes an increased expression of adhesion molecules on the endothelium, the contraction of smooth muscle, and increased vascular permeability [103]. It also stimulates the release of inflammatory mediators. In AA, C5a with its receptor plays an essential role in ANCA-mediated neutrophil recruitment and activation [104]. CD62L is a cell surface protein that plays a crucial role in the adhesion and migration of leukocytes, including neutrophils, which can contribute to leucocyte endothelial migration [105]. CD11c is an integrin present in dendritic cells, monocytes, macrophages, and neutrophils, and is associated with antigen presentation [106]. Although there is no indication of changes in the expression of these antigens in AAV patients, they still may mediate antibody-dependent vascular injury.

### 6.2. The Role of Monocytes in AAV

In healthy individuals, monocytes express low levels of ANCA antigens on their surface [24]. However, in response to pathogen-associated molecular patterns (PAMPs) and danger-associated molecular patterns (DAMPs), the surface expression of ANCA antigens can be upregulated during infection, which makes them available for ANCA antibodies. Indeed, Hattar et al. [107] demonstrated that the pre-incubation of monocytes with PR3-ANCA enhanced the production of interleukin 8 (IL-8), tumor necrosis factor-alpha (TNFα), and IL-6 in response to lipopolysaccharide (LPS) and lipoteichoic acid (LTA), both of which are components of the bacterial cell wall and, as such, serve as PAMPs. This response was associated with the upregulation of the CD14 antigen, a receptor detecting LPS, and a co-receptor of TLR4, suggesting that infections may promote AAV development.

Monocytes can also respond to ANCA by producing ROS [108]. However, a notable reduction in ROS production is observed in AAV patients. Moreover, decreased ROS production correlates with disease activity; patients in remission show higher ROS production than those with an active disease. Furthermore, PMNs from MPA patients showed a lower oxidative burst capacity than cells from GPA and EGPA patients [102].

Monocytes are categorized into three subsets based on the expression of CD14 and CD16 antigen (Fc gamma receptor III): classical (CD14++CD16−), intermediate (CD14++CD16+), and non-classical (CD14+CD16++). Tarzi et al. [109] demonstrated that monocytes of AAV patients with an active disease showed an increased expression of CD14 antigen compared to those in remission, without any changes in proportions between these three subsets. The expression of CD14 was strongly correlated with the expression of ANCA antigens, particularly PR3. Additionally, Matsumoto et al. [110] observed a higher proportion of intermediate monocytes in AAV patients compared to healthy people, regardless of the underlying cause of AAV. AAV patients also exhibited elevated levels of CD62L in monocytes compared to healthy individuals [102]. Moreover, an increased expression of CD11b and CD64 (a high-affinity Fcγ receptor) was observed in monocytes from patients with active GPA, suggesting heightened monocyte activation [111]. Furthermore, these patients demonstrated an increased expression of integrins such as CD29, CD18, CD11a, and CD11b, which are crucial for leukocyte–endothelium interaction and the process of leukocyte diapedesis [112].

### 6.3. Eosinophils as Essential Cells in EGPA

In the case of EGPA, the type of cells involved in inflammatory processes depends on the presence of ANCA [113]. While, in ANCA-positive EGPA, similar to GPA and MPA, neutrophils play an important role, in ANCA-negative EGPA, eosinophils are the predominant immune cells driving inflammation [114]. Eosinophil activation in ANCA-negative EGPA is influenced by type 2 innate lymphoid cells (ILC2) and Th2 cells, which produce IL-5 [115]. IL-5 is a key cytokine responsible for the survival, activation, and recruitment of eosinophils [116]. The pivotal role of IL-5 in EGPA pathogenesis is supported by the clinical efficacy of mepolizumab, a humanized monoclonal antibody targeting IL-5, which prevents eosinophil activation and reduces inflammation [117]. In recognition of its therapeutic benefits, mepolizumab was approved by the US Food and Drug Administration (FDA) in 2017 for the treatment of EGPA.

Upon activation, eosinophils release their granule contents, which include a variety of cytotoxic proteins that contribute to tissue damage and disease progression. These proteins include the major basic protein (MBP), eosinophil cationic protein (ECP), eosinophil peroxidase (EPO), and eosinophil-derived neurotoxin (EDN). Their combined effects lead to cellular damage, endothelial dysfunction, and organ involvement, particularly in the lungs and heart [114]. Moreover, recent studies show that eosinophils release eosinophil extracellular traps (EETs) [40] that can be observed in inflamed tissues in the course of EGPA [118]. EETs, similar to NETs, contain mitochondrial DNA and granule proteins, as mentioned above, and galectin-10. Galectin-10 forms Charcot–Leyden crystals that persist in tissues where they activate inflammasome and sustain inflammation [119].

### 6.4. Importance of DCs

In the vasculitis granuloma, neutrophils and monocytes containing ANCA-specific antigens are surrounded by antigen-presenting cells (APCs), T cells, and B cells [120]. DCs appear to play a central role in the pathogenesis of AAV, as they can drive an inflammatory response to autoantigens by presenting them. The dysregulated activity of circulating DCs has been closely linked to disease progression.

After taking up antigens from neutrophil extracellular traps (NETs), DCs become activated and can initiate autoimmune inflammation and ANCA production [92]. NET-induced DC activation is characterized by an increased TLR4 surface expression and enhanced release of pro-inflammatory cytokines [121]. As TLR4 is responsible for recognizing damage-associated molecular patterns (DAMPs), its expression abnormalities may significantly impact inflammation and tissue damage in the course of AAV. In the experimental animal model, mice immunized with DCs loaded with NETs showed an increase in serum ANCA [92]. The induction of ANCA was accompanied by glomerulonephritis or tubulointerstitial nephritis with vascular neutrophilic infiltration.

Increased spontaneous NET production and the upregulation of the TLR4 receptor have been reported in patients with active MPO-AAV. Interestingly, it was linked with the decreased expression of TIM-3, a co-inhibitory receptor that regulates the T-cell immune response. Su et al. confirmed the regulatory role of TIM-3 in the NET-mediated DC activation in AAV by introducing a TIM-3 blockade in animal experiments [121].

The reduced numbers of circulating DCs (DCs) [122] and plasmacytoid DCs (pDCs) are a commonly reported feature of patients with an active disease [123]. Braudeau et al. [124] demonstrated that conventional DCs (cDCs) from patients with GPA and MPA in the active stage produce significantly lower levels of IL-12 and IL-23p40 following stimulation via TLR3, 4, or 7/8, compared with those from patients in remission or healthy individuals. No such differences were observed in plasmacytoid DCs (pDCs) activity. However, pDCs from MPO-ANCA patients with rapidly progressive glomerulonephritis exhibited a reduced ability to produce IFN-α upon stimulation. This alteration in pDC function was described as a long-term phenomenon, as it persisted even after several years of AAV treatment. The authors attempted to explain the reduced number of circulating pDCs by the migration of this subpopulation to the kidneys, as revealed by an immunohistological analysis [123]. Wilde et al. [125] also reported the presence of both mature (CD208) and immature (CD209) DCs in renal biopsies from AAV patients, emphasizing the critical role of these cells in the inflammatory process.

The study by Tsurikisawa et al. reported that monocyte-derived DCs may be associated with the activity of EGPA, as this group of patients exhibited higher percentages of CD83+ DCs in remission. The elevated CD83+ DCs showed a positive correlation with naturally occurring (nTreg) and inducible Tregs (iTreg), thus implicating a plausible way to induce remission in patients with EGPA [126]. Other literature data also highlight the therapeutic role of DCs in AAV management, including MPO-presenting tolerogenic DCs. However, further studies are required to confirm the precise mechanisms of DCs’ action in AAV and the potential effectiveness of DC-based therapy.

### 6.5. Lymphocytes in AAV

The loss of immunological tolerance towards PR3 or MPO, key autoantigens in AAV, is fundamental to the disease’s development. This breakdown in tolerance is perpetuated by a positive feedback loop of autoreactivity, wherein primed neutrophils or eosinophils release autoantigens while causing vascular damage. The deposition of these autoantigens at injury sites makes them accessible for APCs, which further facilitates antigen recognition by autoreactive T cells. These activated T cells then mediate additional tissue damage. Moreover, AAV patients exhibit a dysfunction of regulatory T cells (Tregs), which exacerbates the loss of tolerance and the emergence of pathogenic ANCA responses.

The course of the immune reaction in AAV, in particular, the type of cells involved in the process of tissue destruction, largely depends on the type of T cells. Naïve helper T cells (Th cells), depending on the cytokines produced by DCs, can differentiate into various subpopulations, including Th1, Th2, Th17, and Tregs (regulatory T cells). This is important in EGPA, where, depending on the type of Th cells, other mechanisms are involved in the development of tissue inflammation. In ANCA-positive EGPA, Th1 and Th17 dominate [127]; they release pro-inflammatory cytokines that activate neutrophils [128] and B cells [129]. The activated neutrophils then expose MPO, and, less frequently, PR3, which is recognized by ANCA. The binding of MPO-ANCA causes NET formation and the release of ROS and proteolytic enzymes that trigger endothelial damage [93]. Meanwhile, in ANCA-negative EGPA, Th2 cells and their cytokines, especially IL-5, play a central role in tissue destruction [130]. As mentioned above, IL-5 is responsible for priming eosinophils to produce enzymesm causing tissue damage, especially in the lungs and heart.

In GPA, the type of Th cells involved in pathogenesis determines whether the disease is localized or generalized [130,131]. In GPA limited to nasal granulomatous lesions, IFN-γ, a marker of the Th1 subset, dominates [105], while, in generalized disease, a high IL-4 expression was observed, suggesting Th2 involvement [132]. The type of Th response is also associated with the disease phase; several authors demonstrated that GPA remission is characterized by Th2 dominance [133,134]. Less is known about T-cell polarization in MPA but it was suggested that it is rather associated with Th1 dominance, especially in patients with the active phase of the disease [135].

Additionally, several studies have highlighted the altered immune cell composition in AAV patients. For instance, AAV patients exhibit increased proportions of activated T cells with HLA-DR [116] or CD25 [136,137] expression compared to healthy controls. In GPA patients, the expression of CD28 antigen, which is an essential co-stimulatory molecule, is significantly decreased in T cells [138]. T cells without any CD28 expression present in those patients are a source of pro-inflammatory cytokines [139] and have the killer cell phenotype [140].

A significant expansion of Th17 cells and a decrease in Tregs have been demonstrated in AAV patients [141]. Th17 expansion may be driven by activated monocytes through TLR ligand interactions, a phenomenon observed in other autoimmune diseases [142]. Increased frequencies of Th17 have been reported in EGPA patients during disease relapse [143]. In GPA, the presence of Th17 was independent of disease activity [144]. The suppressive function of Tregs is significantly impaired, especially during an active disease, as reported by Kitching et al. [42], but tends to normalize during periods of remission [134].

The role of natural killer (NK) cells in AAV remains uncertain [145], with some studies showing a reduction in NK cell numbers or percentages, while others report an increase. Some authors found that NK cells from GPA patients are characterized by a higher expression of the early activation marker CD69, suggesting an altered NK cell response in AAV [146].

B cells are integral to the pathogenesis of AAV, primarily through their role as precursors of antibody-secreting plasma cells [147]. These cells regulate immune responses by inhibiting T-cell proliferation and the production of pro-inflammatory cytokines, such as interferon-γ (IFN-γ), TNF-α, and IL-17 [148]. Additionally, B cells function as APCs, initiating T-cell responses by providing costimulatory signals and producing cytokines and growth factors [149]. The significance of B cells in AAV pathogenesis is further supported by the success of B-cell depletion therapies, such as rituximab (RTX). RTX is as effective as CYC treatment in inducing remission in severe AAV cases and maybe even more effective in preventing relapses [150,151]. Importantly, the reappearance of B cells and the return of ANCA positivity following RTX treatment are associated with a higher risk of AAV relapse [152]. Specific changes in the frequencies of B cells were also reported, including a decrease in the percentage of transitional B cells and an increase in switched memory B cells and plasmablasts, reflecting an adaptive immune response that sustains the inflammatory process in AAV.

## 7. Conclusions

The comparison of all three AAV subtypes is summarized in Table 2. As shown, although considerable progress has been made in elucidating the genetic and cellular mechanisms underlying AAV, many critical aspects of its pathogenesis remain poorly understood. In particular, the role of epigenetic regulation in the initiation and progression of the disease is still largely unexplored. Additionally, the contribution of specific immune cell populations—such as dendritic cells (DCs) and natural killer (NK) cells—remains insufficiently characterized, despite the growing evidence of their involvement in other autoimmune and inflammatory conditions. A deeper understanding of these factors could significantly enhance our ability to identify biomarkers for disease activity and prognosis, as well as uncover novel therapeutic targets. Ultimately, continued research into these underexplored areas holds promise for the development of more precise and effective treatment strategies for patients with AAV.

## Figures and Tables

**Table 1:** ANCA types.

	p-ANCA	c-ANCA
Autoantigens	MPO, cathepsin G, elastase, lactoferrin, and lysozyme	proteinase 3
Staining pattern	perinuclear	cytoplasmic
AAV	MPA, EGPA	GPA

**Table 2:** Comparison of GPA, MPA, and EGPA.

	GPA	MPA	EGPA
Clinical presentation	Respiratory tract involvement, and ocular symptoms (uveitis, ocular ulcers, episcleritis, and scleritis)	Glomerulonephritis, pulmonary capillaritis, skin, nerves, and gastrointestinal tract involvement	Respiratory tract involvement (asthma as a typical early feature), and neurological symptoms
Granulomas	Present	Not present	Present
The main type of ANCA	PR3-ANCA	MPO-ANCA	MPO-ANCA
Percentage of ANCA(−) patients	10%	10%	50%
Main effector cells	Neutrophils	Neutrophils	Neutrophils in ANCA(+), and eosinophils in ANCA(−)
T-cell polarization	Th1 (localized disease), and Th2 (generalized disease)	Th1	Th1, Th17 in ANCA(+), and Th2 in ANCA(−)
